# Influence of epidural anesthesia on the hemocoagulation disorders and quantity of septic complications in patients with acute necrotizing pancreatitis

**DOI:** 10.1186/cc12292

**Published:** 2013-03-19

**Authors:** O Tarabrin, S Shcherbakov, A Tkachenko, O Kushnir, I Grychushenko

**Affiliations:** 1 Odessa National Medical University, Odessa, Ukraine

## Introduction

According to many authors, acute necrotizing pancreatitis (ANP) still remains one of the difficult problems of abdominal surgery. The complexity of the pathogenesis of the disease, features of the pancreas pathomorphology, abdominal hypertension, and high mortality (30 to 70%) necessitate a search for new ways to treat this disease.

## Methods

The study was conducted in 44 patients with ANP, who were divided into two groups according to type of analgesia: epidural or opioids. Patients from the first group (*n = *23) had epidural analgesia by ropivacaine 6 to 14 mg/hour during 7 to 10 days, and from the second group (*n = *21) opioid analgesia by trimeperidine 20 mg three times a day during the same period. We monitored the level of septic and thrombohemorrhagic complications by clinical and instrumental data, during the month after treatment starting. The hemostatic system was evaluated using indicators of hemoviscoelastography (Mednord-01M analyzer).

## Results

It was found that all patients with ANP initially have hypercoagulation and fibrinolysis inhibition. Levels of hemostatic disorders correlate with the level of septic complications, treatment in the ICU, and mortality. In the first group we noted a deep vein thrombosis, two pneumonia, seven pseudopancreatic cysts and abscesses, two deaths and time of stay in the ICU as 15.4 days. In the second group: three cases of deep vein thrombosis, four pneumonia, 10 pseudopancreatic cysts and abscesses, two episodes of gastroduodenal bleeding, five deaths and time of stay in the ICU as 27.8 days.

## Conclusion

Using epidural anesthesia in patients with ANP reduced the number of septic complications on 36.6%, and reduced the mortality rate from 23.8% (second group) to 8.7% (first group). We think that violations of blood coagulation and microcirculation are the basis for ischemia, necrosis in tissues and septic complications. Epidural analgesia is an effective method to decrease the level of septic and thrombohemorrhagic complications and mortality in ANP patients.

